# The role of motion management and position verification in lymphoma radiotherapy

**DOI:** 10.1259/bjr.20210618

**Published:** 2021-09-22

**Authors:** Marianne Aznar, Georgios Ntentas, Marika Enmark, Stella Flampouri, Peter Meidhal Petersen, Umberto Ricardi, Mario Levis

**Affiliations:** 1 Division of Cancer Sciences, Faculty of Biology, Medicine and Health, University of Manchester, Manchester, UK; 2 Nuffield department of population health, University of Oxford, Oxford, UK; 3 Department of Medical Physics, Guy’s and St Thomas’ NHS Foundation Trust, London, UK; 4 Department of Hematology, Oncology and Radiation Physics, Skåne University Hospital, Malmö, Sweden; 5 The Skandion Clinic, Uppsala, Sweden; 6 Department of Radiation Oncology, Emory University, Atlanta, GA, USA; 7 Department of Oncology, Rigshospitalet, University of Copenhagen, Copenhagen, Denmark; 8 Department of Oncology, University of Torino, Turin, Italy

## Abstract

In the last decades, the substantial technical progress in radiation oncology offered the opportunity for more accurate planning and delivery of treatment. At the same time, the evolution of systemic treatment and the advent of modern diagnostic tools allowed for more accurate staging and consequently a safe reduction of radiotherapy (RT) target volumes and RT doses in the treatment of lymphomas. As a result, incidental irradiation of organs at risk was reduced, with a consequent reduction of severe late toxicity in long-term lymphoma survivors. Nevertheless, these innovations warrant that professionals pay attention to concurrently ensure precise planning and dose delivery to the target volume and safe sparing of the organs at risk. In particular, target and organ motion should be carefully managed in order to prevent any compromise of treatment efficacy. Several aspects should be taken into account during the treatment pathway to minimise uncertainties and to apply a valuable motion management strategy, when needed. These include: reliable image registration between diagnostic and planning radiologic exams to facilitate the contouring process, image guidance to limit positioning uncertainties and to ensure the accuracy of dose delivery and management of lung motion through procedures of respiratory gating and breath control. In this review, we will cover the current clinical approaches to minimise these uncertainties in patients treated with modern RT techniques, with a particular focus on mediastinal lymphoma. In addition, since uncertainties have a different impact on the dose deposition of protons compared to conventional x-rays, the role of motion management and position verification in proton beam therapy (PBT) will be discussed in a separate section.

## Introduction

In the last decades, radiotherapy (RT) for Hodgkin lymphoma (HL) has become increasingly more targeted. The irradiation fields are limited to the involved sites or involved nodes and prescribed doses have been significantly reduced compared to the past.

Multimodality treatment combining a short course of chemotherapy (2–4 cycles) and involved node radiation therapy (INRT) has been shown to be safe and effective^1,2^ in early-stage HL patients. The INRT concept, in which only the lymph nodes showing disease involvement before chemotherapy are irradiated with a small margin for set-up variation, was introduced by the European Organisation for Research and Treatment of Cancer (EORTC) in 2006.^3^ In 2014, the International Lymphoma Radiation Oncology Group (ILROG) introduced involved site radiation therapy (ISRT), a slightly larger volume intended to allow for uncertainties in image registration encountered when pre-chemotherapy imaging is “sub optimal” according to INRT requirements, for example, not acquired in the same position as the RT planning CT scan. Both INRT and ISRT concepts require following specific imaging guidelines for accurate disease localisation, for example, co-registration of the baseline pre-chemotherapy positron emission tomography/computed tomography (PET/CT) with the RT planning CT.

As a direct consequence of these changes, the need for accurate dose delivery has increased, both to ensure that the required dose is delivered to the target volume, but also to spare the organs-at-risk (OARs) as much as possible to limit the risk of acute and late radiation-related toxicity. Image guidance plays a fundamental role in ensuring the accuracy of dose delivery. The introduction of frequent (often daily) 3D image guidance has led the gradual reduction of positioning uncertainties, and thereby a trend towards the possibility of reducing the clinical target volume (CTV) to planning target volume (PTV) margins in clinical practice. Although the 2014 ILROG guidelines recommended CTV to PTV margins of 10 to 15 mm,^4^ recent reports suggest that tighter margins (<10 mm) can be used without a detriment in outcomes with appropriate use of daily image guidance.^5^ Although daily imaging comes with additional dose (between 30 and 80 mGy per scan for standard CBCT protocols), it has been suggested that this exposure is compensated by the reduction in target volume.^6,7^

The reduction of irradiated volumes requires increased attention to the residual sources of uncertainty in the treatment pathway. In addition to positioning uncertainties, two sources of variation take a greater importance as RT delivery becomes more targeted: i) image registration between pre-chemotherapy PET/CT and RT planning CT and ii) respiratory motion. This has been translated into additional efforts to address uncertainties (*e.g.* variations in treatment position throughout the treatment course, as well as the effect of respiratory motion on mediastinal and abdominal targets).

In this review, we will cover the approaches to minimise these uncertainties (grouped under the general term of “motion management”) in INRT and ISRT, with a particular focus on mediastinal HL. In addition, since uncertainties have a different impact on the dose deposition of protons compared to conventional X-rays, the role of motion management and position verification in proton beam therapy (PBT) will be discussed in a separate section.

## Motion management before chemotherapy

### General considerations

Patients treated for lymphoma will experience considerable anatomical changes between staging/ diagnosis and the start of RT, for example, due to considerable shrinkage of the gross tumour volume (GTV) during and after chemotherapy. The process of transferring the pre-chemotherapy GTV to the RT planning scan and adapting it to the post-chemotherapy anatomy is challenging: uncertainties in this process could result in a risk of tumour geographic miss or in larger volumes of healthy tissues being irradiated, with a potential increase in long-term complications depending on the lymphoma location.

High-quality pre-chemotherapy imaging is, therefore, fundamental to enable accurate contouring of the target volume at the pre-treatment stage. Baseline PET/CT and contrast-enhanced CT scans are both essential, and not mutually exclusive, for accurate staging and delineation of the treatment volumes. The CT scan must always be contrast-enhanced and is particularly useful in the definition of small lymph nodes or lymphoma sites not evident in the PET/CT scan. It is also essential to offer additional imaging such as magnetic resonance imaging when required to clarify disease involvement, in particular sites like the brain or the head and neck region. Moreover, clinicians must be careful in the interpretation of imaging and in differentiating organ displacement from infiltration (*e.g.* pleural and pericardial effusion *vs* tumour infiltration or lung atelectasis *vs* parenchymal infiltration).

### INRT conditions

**Figure 1.**
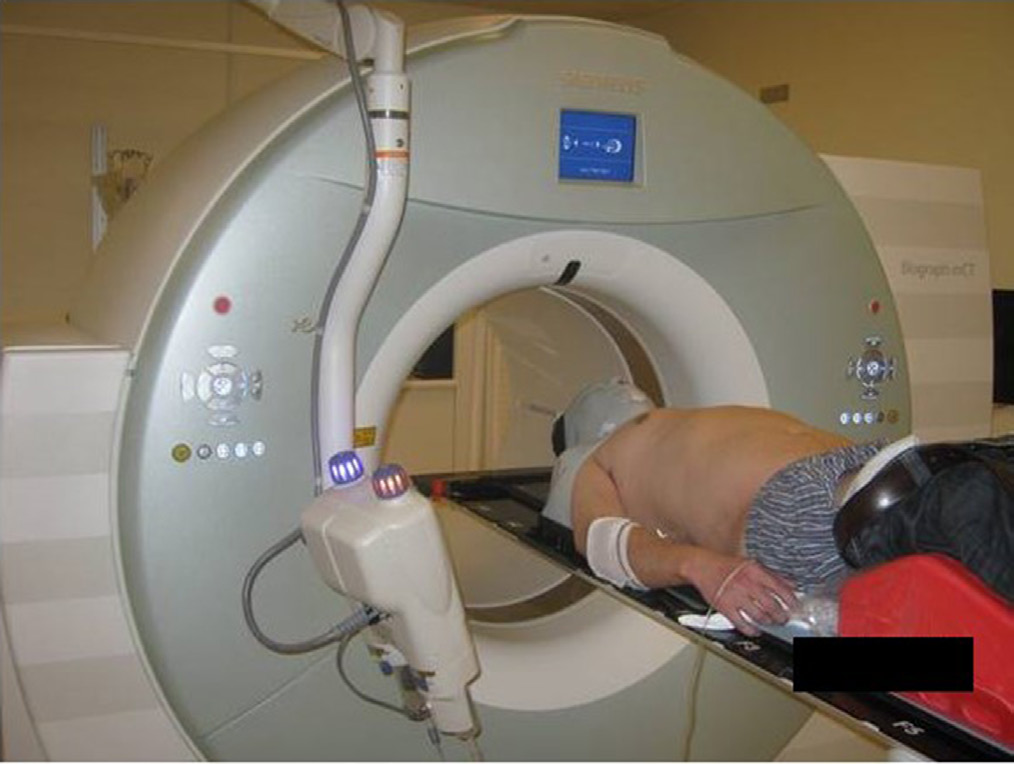
Example of pre-chemotherapy image acquisition satisfying INRT conditions. The PET/CT scanner is fitted with a flat table top. The patient position mimics that of the anticipated treatment position for radiotherapy. Here, the patient is scanned with arms down and a thermoplastic mask covering the head and shoulders. A knee pad (red) is used to increase comfort. This approach necessitates a close collaboration between the nuclear medicine and the radiotherapy teams (for example, radiotherapy radiographers may be present during pre-chemotherapy PET/CT alongside nuclear medicine technologists).

INRT requires optimal pre-chemotherapy imaging (both PET/CT and contrast-enhanced CT scans) in the treatment position to allow an accurate image registration with post-chemotherapy and planning scans.^3,8^ An example of pre-chemotherapy patient position satisfying INRT conditions is illustrated in Figure 1. The patient lies on a flat-table top, in a thermoplastic mask, with arms down. The inclinations of the board, arm rest position, and other details are noted in order to reproduce the position as accurately as possible post-chemotherapy. It should be noted that this approach requires excellent communication between nuclear medicine and/or radiology technicians and RT radiographers and may not be logistically feasible in many treatment centres.

In addition to patient positioning, INRT guidelines recommend that scans should be evaluated by a radiologist and encourage the use of immobilisation devices as well as “respiratory gating” to match the RT delivery conditions as closely as possible.^3^ The most common form of respiratory gating in HL is Deep Inspiration Breath Hold (DIBH).^9^ In addition to motion management, a key role of DIBH is to increase the distance between the irradiated volume and the heart, thereby reducing cardiac exposure.^10,11^

**Figure 2.**
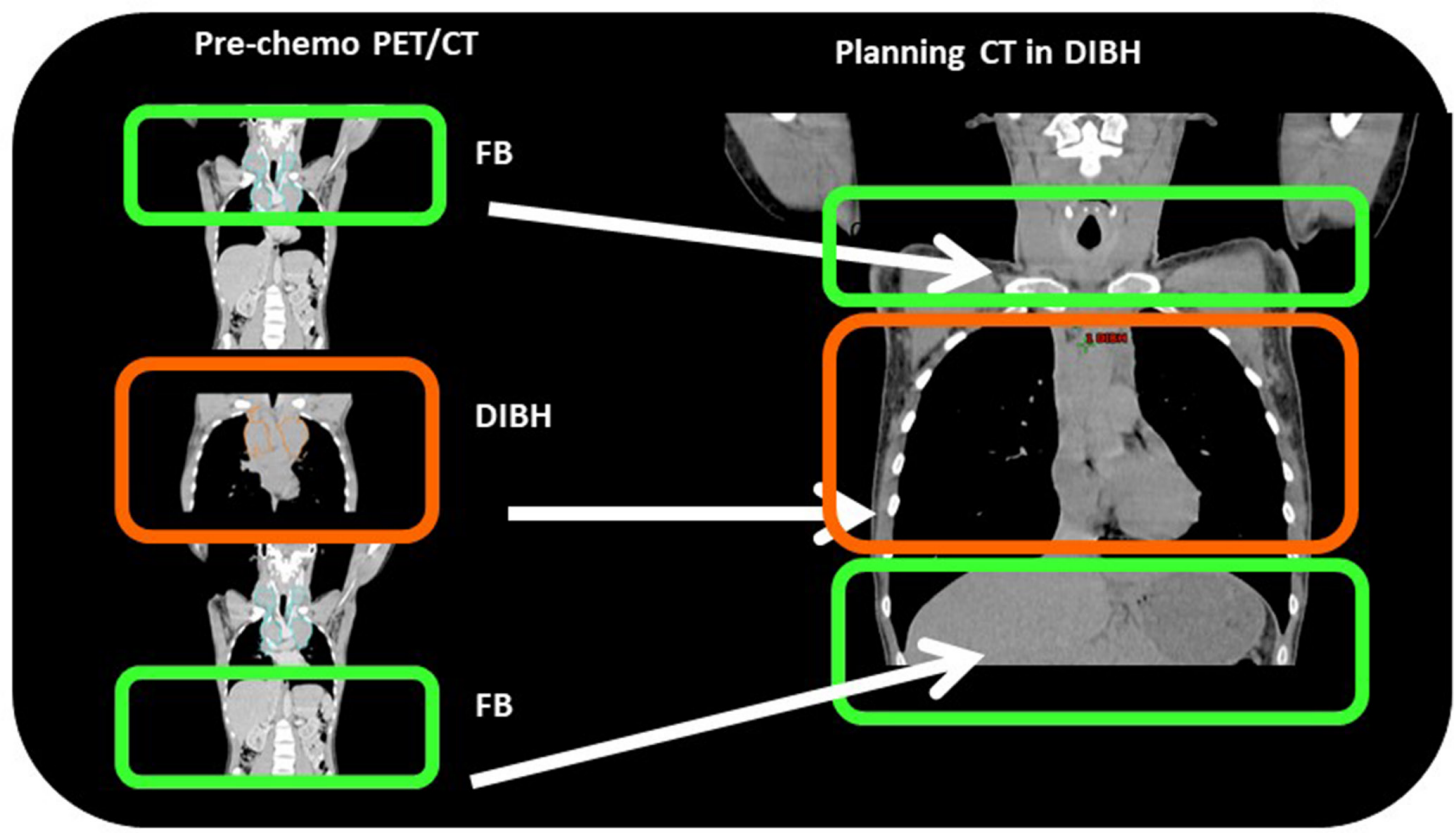
Illustration of the image registration strategy between the pre-chemotherapy PET/CT in free breathing and deep inspiration breath hold (DIBH), and the radiotherapy planning CT in DIBH. In the pre-chemotherapy scan, only a limited field of view (PET bed) is acquired in DIBH, for example, over 1 breath-hold (for the CT) and 6 breath-holds (for the PET). Each breath-hold lasts about 20 s.

The INRT concept implies that, if RT is delivered in DIBH, pre-chemotherapy imaging should be also performed in DIBH if possible. For example, at Rigshospitalet, Copenhagen, Denmark where the nuclear medicine department and RT department share common facilities, mediastinal HL patients who will likely need combined modality treatment receive a full-body pre-chemotherapy fluorodeoxyglucose (FDG) PET/CT scan while breathing freely, immediately followed by a DIBH PET/CT scan. The DIBH PET/CT scan is limited to a single field of view acquired over seven breath-holds of 20 s each (Figure 2). Should the patient later be referred for RT, these images can be registered with a treatment planning CT (see Milgrom et al,^12^ in this issue for more information on the registration process). Other centres, such as Guy’s and St Thomas’ Hospital in London, are now implementing similar methods. If a PET/CT cannot be performed in DIBH, an alternative approach could be to acquire a pre-chemotherapy CT scan in DIBH, and a PET/CT in free breathing.

DIBH guidance equipment can be implemented on dedicated RT CT scanners (and often, if required, on PET/CT scanners) and in the treatment room. Using the same equipment during both pre-treatment imaging and treatment delivery, minimises the risk of systematic variations in patient position and in breath-hold level between the staging scan and the RT planning scan.

The acquisition of PET/CT in DIBH has been received positively by clinical oncologists and radiologists, who have the experience that the procedure increases their confidence in transferring tumour volumes from the pre-chemotherapy to the RT planning scan. It is important to note that evidence of reduced uncertainty, or impact on contouring is difficult to obtain, since it is challenging to isolate the impact of PET/CT in DIBH from other sources of intra- and interobserver variation. In addition, the procedure requires an additional 15 min on the PET/CT scanner and the additional acquisition of a short DIBH CT (for attenuation correction of the PET DIBH).

### ISRT conditions

The ISRT principle was introduced in 2014 by ILROG as an alternative approach to INRT for when optimal pre-chemotherapy imaging is not available, for example,. when the patient position during scanning differs between baseline and treatment. For example, PET imaging is often performed with arms up, while RT may need to be delivered with arms down, either to allow the use of specific immobilisation devices or to minimise the dose to some OARs (*e.g.* breast tissue). In this scenario, image registration will be more challenging. Recent ILROG guidelines^13^ describe most clinical scenarios for ISRT application.

Clinical judgement and expertise in the treatment of lymphoma patients are fundamental to properly apply the ISRT concept. Nevertheless, a standardisation of this planning process is still lacking and a certain level of discrepancy in the definition of target volume is still observed, even among experienced radiation oncologists^14,15^

## Motion management in radiotherapy planning

### General considerations

A first important consideration is whether RT is delivered as primary treatment or as part of a combined modality approach. In the first case, which occurs less frequently and is limited to selected clinical scenarios, the GTV is easily identifiable on the planning CT scan. In the latter case, patients receive several cycles of chemotherapy (frequently ≥4) that markedly affect the volume and shape of the GTV. When the patients come back to the radiation oncology department for their RT planning CT, several factors must be taken into consideration to compensate the effects of systemic therapy.

**Figure 3.**
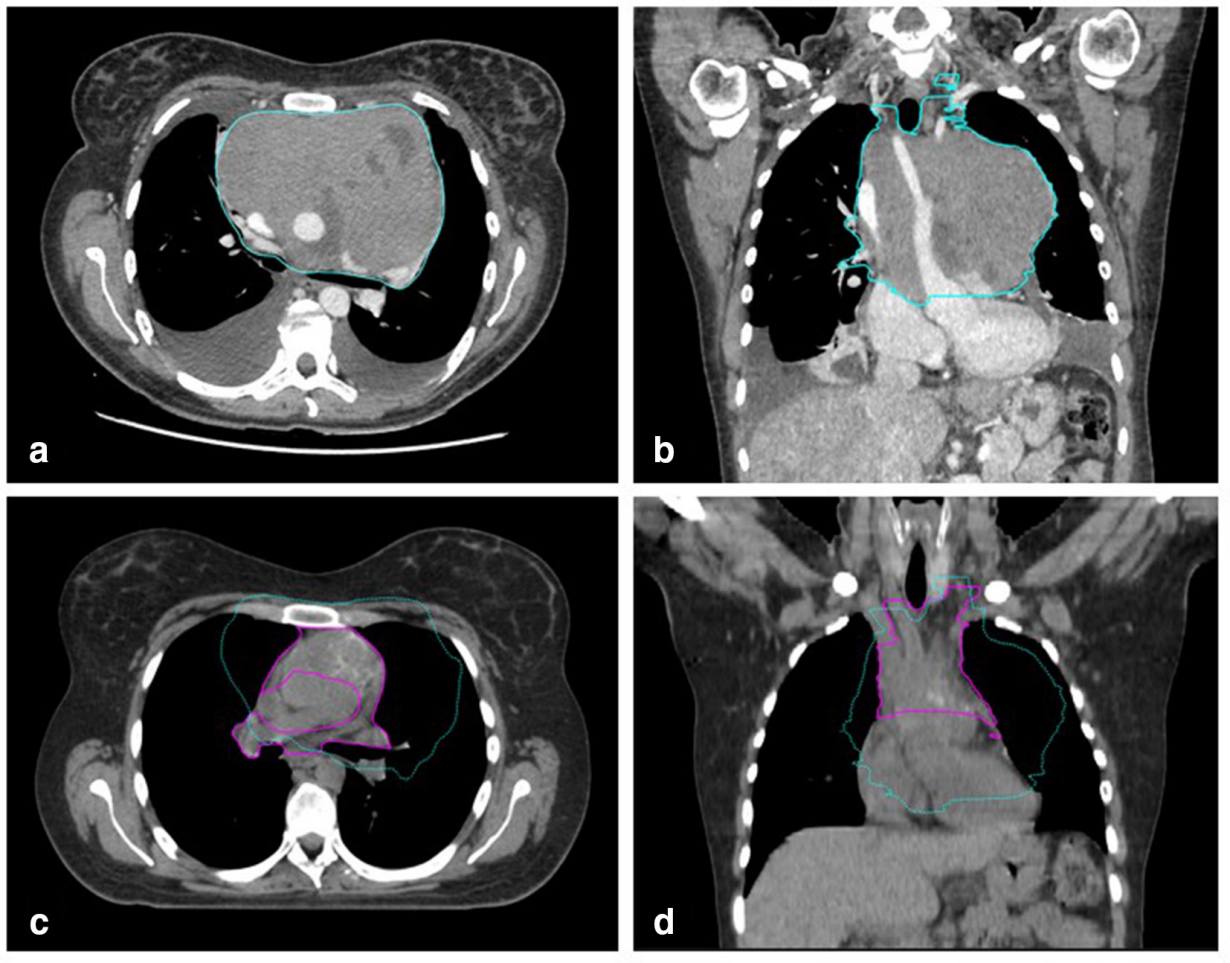
Contouring process according to the ISRT principles. Pre-chemotherapy GTV is contoured on the baseline radiologic exams (blue volume in a and b). Planning CT scan is then co-registered with pre-chemotherapy exams in order to translate the GTV volume (blue volume in c and d). The same GTV volume then needs to be modified to generate the CTV (pink volume in c and d), in order to exclude healthy organs such as muscles, vessels lungs and heart that were clearly uninvolved at diagnosis.

In fact, response to chemotherapy may cause significant tumour shrinkage and displacement of normal structures, particularly in patients with mediastinal or abdominal bulky lesions at baseline. The anatomy can be greatly modified by the changes in shape and position of the healthy OARs. Therefore, the GTV needs to be modified to generate the CTV, in order to exclude healthy OARs and structures (such as lungs, breast, heart and surrounding muscle) that were clearly uninvolved at diagnosis (Figure 3). Moreover, patient positioning may differ between baseline and planning CT scans. In particular, arms elevation (up or down) and lung inflation (free breathing or DIBH) may complicate CTV delineation. A deformable image registration between diagnostic and planning scans is recommended to limit these uncertainties and to prevent the need of an excessive compensatory enlargement of the CTV volume. However, since deformable image registration algorithms do not always handle shrinking volumes well, a careful review of the fused images is necessary.

Subclinical disease involvement is another consideration. It is related to many clinical features such as histology, stage, anatomical site, nodal or extranodal location as well as type and dosage of any previous therapy. The extension of the treated volume should take into account all of these parameters. As an example, CTV delineation may be more generous when modern RT (INRT or ISRT) is not preceded by any systemic therapy, as in early-stage indolent B-cell and lymphocyte predominant HL, to encompass the suspected contiguous sites of subclinical disease.

Tumour coverage must be counterbalanced against incidental radiation dose to surrounding OARs, which are influenced by the prescribed dose and the location and extension of the disease. Additionally, the associated toxicity risk which does not only depend on radiation doses but also on other risk factors such as age at treatment, sex, smoking status, obesity and other traditional risk factors, including chemotherapy combinations, must also be taken into account. Young patients are known to have a higher risk of developing serious complications.^16–19^ The most challenging cases are usually mediastinal lymphoma patients with bulky disease, where dose constraints for some relevant OARs might be exceeded. In this case, clinical judgement is fundamental to find the best compromise between tumour control and risk of radiation-related late effects. Contouring of all OARs (including the heart substructures) is the first step for a decision process, oriented to prioritise sparing of structures considered more sensitive through treatment planning optimisation.^20^ However, it is worth noting that organ motion during delivery, (in particular of lung and heart) may further complicate an accurate estimation of the dose received by the healthy tissues.

### Managing respiratory motion

#### Treatments in free breathing

RT planning on a free breathing CT scan has been the main method for mediastinal and abdominal lymphoma patients since the early 2000s. However, it was recognised that respiratory motion would affect the target volumes; therefore, CTV to PTV margins were often expanded by 5 mm in the Superior-Inferior (SI) direction to account for breathing.^21^ The widespread use of 4D-CT in other tumour sites such as lung cancer provides a potential to individualise margins in lymphoma patients to account for breathing motion for targets in the chest and upper abdomen. Patient images are acquired throughout the respiratory cycle in order to characterise target volume motion and produce an internal target volume to account for it.

In mucosa-associated lymphoid tissue (MALT) lymphoma, 4D-CT has been used to reduce irradiated volumes, especially when SI motion is over 15 mm.^22^ In contrast, there are few reports on the use of 4D-CT in mediastinal lymphoma (in photon-based RT). This could be explained by the fact that mediastinal targets are generally less mobile than abdominal targets (with the notable exception of cardiophrenic disease), or due to concerns for the additional imaging dose in this patient group. Filippi et al. mention that 4D CT may provide additional information in patients where a larger mediastinal motion is suspected.^23^ In recent years, more institutions report using isotropic margins instead of expanding margins in the SI direction to account for respiratory motion.

With a well-designed image-guidance protocol and careful consideration of all uncertainties, it has been shown that margins in modern RT can be safely reduced compared to the standard (10–15 mm) from earlier guidelines. For example, a recent report from Levis et al suggests that the use of a 5 mm CTV to PTV margins in patients planned with volumetric modulated arc therapy (VMAT) and with daily cone beam CT (CBCT) led to no increase in relapse risk with a median follow-up of 5 years on a cohort of more than 200 HL patients.^24^ The type and frequency of CBCT imaging during RT should be considered when deciding margins, as the smaller the margins the more frequent the imaging should be to ensure adequate coverage of the target volumes. Optimising CBCT scanning parameters can help to further reduce the concomitant imaging dose whilst ensuring adequate tumour coverage.

#### Treatments in deep inspiration breath hold

The role of DIBH in motion management is twofold: for mobile targets, it can reduce the displacement of the target volume; for targets close to a sensitive OAR, the inflation of the lungs can increase the distance between organs and target volume, and facilitate OAR sparing. In mediastinal HL, for example, the rationale for DIBH often focuses on reducing the dose to the heart. It is important to note that the DIBH manoeuvre is subject to variations (between fractions, as well as during a single treatment fraction). Because of these uncertainties, the use of DIBH by itself will not necessarily lead to a reduction of margins compared to free-breathing. Therefore, DIBH-specific uncertainties should be included in the treatment margins.

When treatment is to be delivered in DIBH, the most common approach is to acquire a free breathing scan followed by a DIBH scan during the same simulation session. If i.v. contrast is required, it can be used during the DIBH scan. The free breathing scan is then used as a back-up in case the DIBH plan is not dosimetrically superior to the FB plan, or if the patient is no longer able to perform DIBH during the course of treatment.

**Figure 4.**
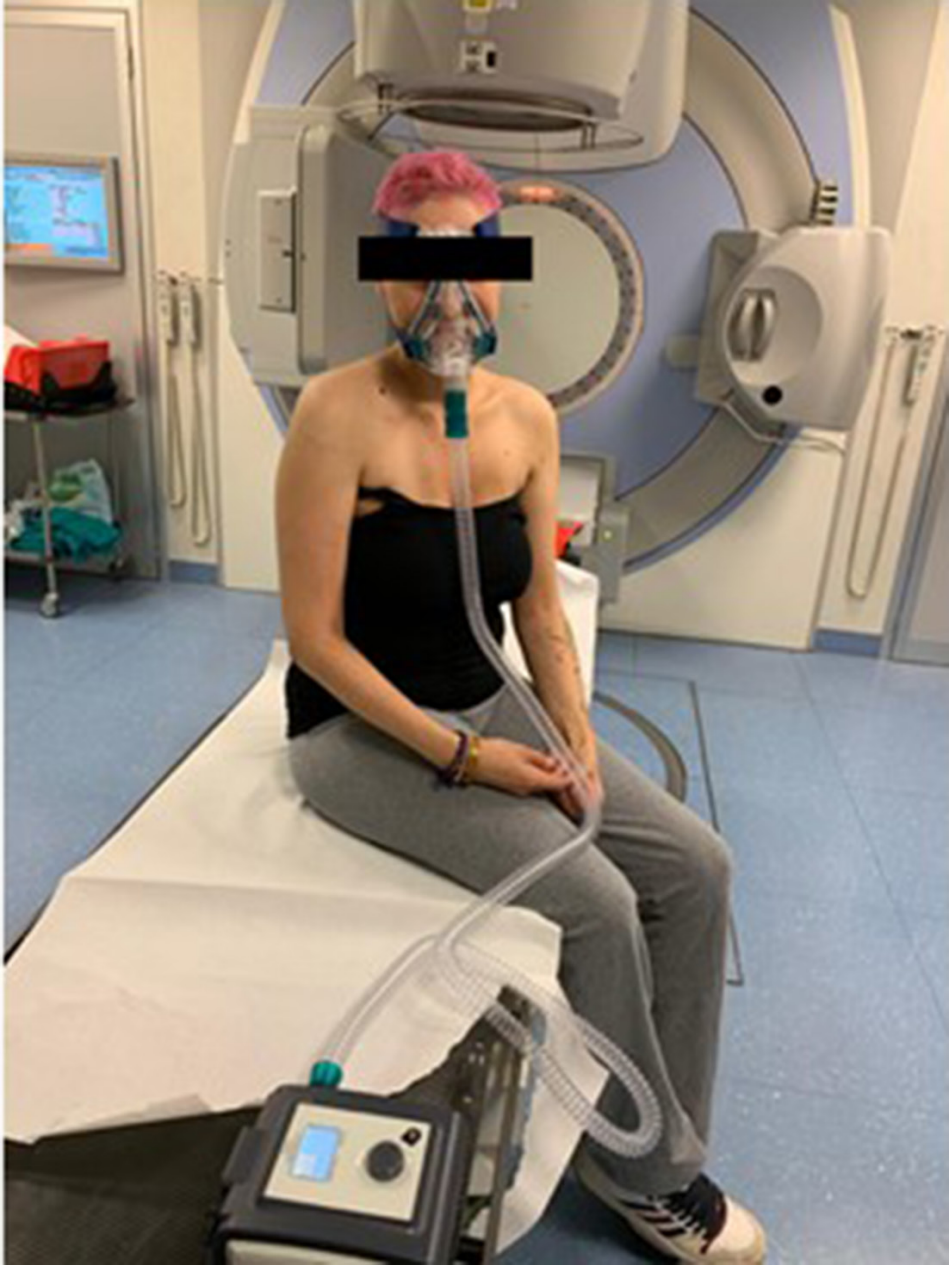
Example of motion management during treatment delivery using a continuous positive airway pressure (C-PAP) device (patient not in treatment position). The mask is fitted over the patient’s face, and airway pressure is regulated through the control box (bottom of the figure).

**Figure 5.**
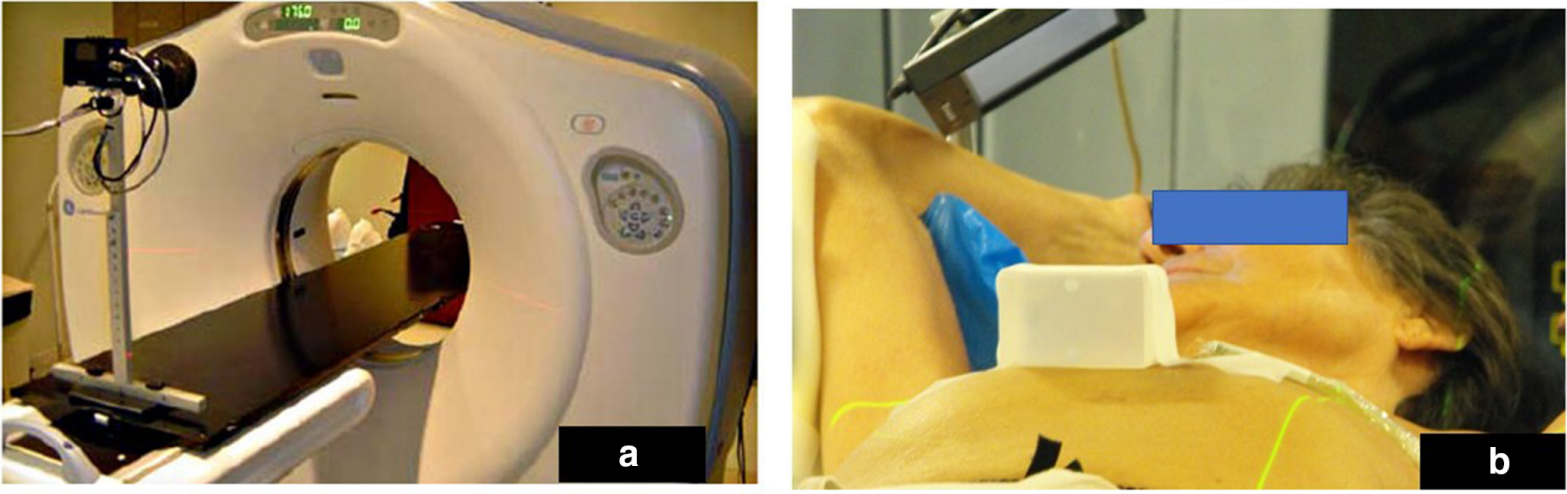
Example of motion management during treatment planning and delivery using a system-based on optical markers (here RPM^®^ from Varian Medical Systems). (a) The scanner is fitted with a flat table, and an infrared camera is positioned at the feet of the patient during scanning (for treatment planning, and for pre-chemotherapy scanning as well in INRT conditions). (b) A marker box is positioned on the chest of the patient, and its displacement is tracked to ensure a reproducible level of inspiration or breath hold.

Supradiaphragmatic patients receive the greatest dosimetric benefits from DIBH. Several studies have demonstrated that DIBH can reduce incidental radiation dose to the heart and lungs compared to FB due to the increased lung volume and the location of the heart, which is pulled caudally during breath hold.^8,10,25,26^ There are many DIBH systems on the market, and innovative solutions to increase patient comfort and reproducibility are constantly being developed. In this review, we will consider two main categories of DIBH systems: (1) guided approaches (*i.e.* direct interventions on the patient’s breathing) and (2) voluntary approaches where the patient’s breathing is closely monitored without direct intervention.^27^ In guided DIBH, devices usually utilise a spirometer that allows active breathing control by monitoring and stopping air flow at a set volume threshold. An alternative is the use of continuous positive airway pressure devices, to gently inflate the lungs (Figure 4). In voluntary DIBH on the other hand, respiratory motion is monitored, and the patient is asked to take a breath hold voluntarily to a certain point that is defined by an external device. This device is often an optical tracking system, and monitoring can be facilitated by tracking markers/reflectors (Figure 5) or projecting visible light on the patient to monitor the patient surface. Voluntary and moderate DIBH techniques were compared in a study on breast patients, which found that they were comparable in terms of positional reproducibility and normal tissue sparing. However, in this patient group, voluntary DIBH was preferred by patients and radiographers as it usually took less time for treatment setup.^28^

In some cases, the RT technique used locally might define which DIBH solution should be used. For example, if non-coplanar beam arrangement is used, such as Butterfly-VMAT,^20,26,29,30^ some moderate DIBH systems might not be feasible as the non-coplanar beams may cause a collision between the gantry and the DIBH equipment. With voluntary DIBH systems, additional attention may need to be paid to the external signal, for example, using an additional camera or additional angles for the optical tracker: this will help monitor the breathing cycle even if couch rotation is needed when non-coplanar beams are used. Another example is that of the recent clinical implementation of integrated MR-Linac systems and their potential in the treatment of lymphoma^31^ where MR-compatible DIBH systems will need to be developed^32^ to maintain this beneficial technique.

DIBH can be helpful in reducing heart motion, although it cannot be completely eliminated. Novel strategies include a detailed contouring of all cardiac structures to be included in the optimisation process of the treatment plan. Moreover, the adoption of expansion margins (“planning risk volumes” or PRVs) could account for heart motion,^33,34^ improving avoidance of the heart and the protection of small and clinically relevant structures such as the coronary arteries.^35^

As with free-breathing treatments, DIBH treatment must consider the type and frequency of on-board imaging during RT when deciding margins.

#### Gastric lymphoma

In contrast with mediastinal HL, where breathing control techniques are now widely used, there are very few reports in the literature about the use of motion management in gastric lymphoma. This may be due to the relatively low number of cases, or to the fact that 4D-CT is more widely used in the abdomen. However, planning studies have shown that breathing controlled RT of the stomach with treatment only in pre-specified respiratory phases reduces doses to the OARs. Additionally, one study showed that DIBH can reduce the dose to liver, heart, lung and spinal cord without compromising the dose to the stomach and surrounding lymph nodes.^36,37^

## Motion management during radiotherapy delivery

### Position verification

As mentioned earlier, the use of more targeted radiation delivery requires optimal immobilisation, for example, using chest boards or thermoplastic masks depending on the location of the target. When target volumes are significantly extended craniocaudally, usually when mediastinal and head and neck nodes are involved, daily multiscanning and immobilisation might be necessary to maintain confidence of patient positioning and target coverage. Immobilisation solutions combining thoracic boards with 5-point head and shoulder thermoplastic masks are widely utilised in this patient category (Figure 1).

Although the dose burden from daily CBCT should be considered, Zhu et al^7^ suggest that the imaging dose is more than offset by the reduction in CTV to PTV margin, and subsequent reduction of treatment dose to OARs. For large volumes, for example, extending from the neck to the lower mediastinum, it may be impossible to visualise the full target volume on position-verification images. In this case, Aristophanous et al^38^ recommend the use of location-specific margins, to account for additional uncertainties in regions which cannot be visualised. Filippi et al. reported excellent control in patients treated for mediastinal HL to 30 Gy using 8 mm CTV to PTV margins, intensity-modulated RT (IMRT) and using daily CBCT or megavoltage MV-CT.^5^ In MALT lymphoma, it was suggested that CTV to PTV margins could be even further reduced with the use of 4D-CBCT.^39^

### Verification of breath-hold level in DIBH treatments

If the treatment is delivered in DIBH, the position verification images should be acquired in DIBH as well. CBCT is usually acquired in DIBH over several short consecutive breath holds. Depending on the integration of the respiratory motion system with the linear accelerator, this acquisition is either automatically gated, or the treatment radiographers may start and stop the image acquisition manually to allow the patient to catch their breath. For fit patients, it is also possible, with appropriate scan settings, to perform the image acquisition in one single breath hold of about 30 s. In systems where gating or pausing the image acquisition during CBCT is not possible, three pragmatic options can be used: (1) to ask the patient to hold one, long breath and accept that some of the acquisition might occur in free breathing, (2) to perform a continuous acquisition over several breath holds separated by short periods of free breathing or (3) to acquire a partial CBCT. One additional advantage of acquiring CBCT in breath hold is that the imaging quality is improved considerably: in lung cancer patients, Josipovic et al^40^ have demonstrated a considerable improvement both in qualitative image evaluation and in terms of registration accuracy.

## Motion management in proton beam therapy

Proton beam therapy, with its unique characteristics of a high dose peak and a sharp fall off at the end of the beam range, has the potential to improve target dose conformity and OAR sparing compared to photon RT and has recently been introduced in the management of lymphomas.^41^ The proton beam range, while inherently conforming to the target, is sensitive to tissue density variations within the entire beam-path. Respiratory motions and deformations can potentially result not only in target position uncertainties but also in spatial variations of the dose distribution.

Pencil beam scanning (PBS), currently the most advanced and commonly used proton delivery technique, adds to the difficulty by painting a moving target with a scanned narrow proton beam.

The interference between target motion and dynamic beam delivery, referred to as “interplay effect” can degrade the dose distribution by creating dose inhomogeneities within the target. Hence, mitigation techniques are necessary to ensure that the delivery of dose to the target is robust even in the presence of respiratory motion.

Free breathing PBT treatments are based on 4D-CT. As in photon treatments, DIBH, guided or voluntary, has the potential to reduce the magnitude of motion. However, when DIBH is used with protons, more stringent procedures must be in place to reduced detrimental uncertainties and variations. Multiple breath-hold scans are acquired^37^ to establish sufficient margin and breath-hold reproducibility under conditions simulating the length and process of treatment. Assessment of target displacement from multiple breath-hold scans is similar to 4D-CT-based motion evaluation.

**Figure 6.**
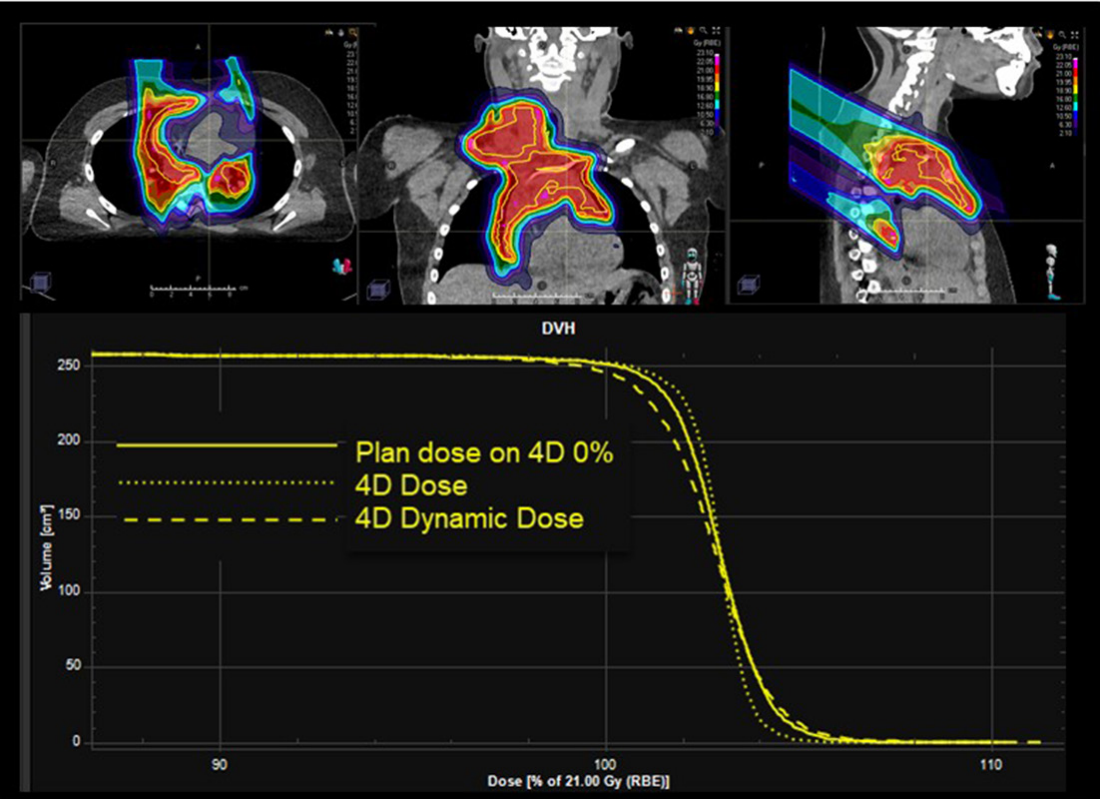
Proton beam therapy for Hodgkin lymphoma. Top row: dose distribution over the clinical target volume (CTV) (outlined in yellow) in the lower neck and mediastinum. Bottom panel: dose volume histogram for the CTV (solid line) showing the effect of motion (dotted and dashed).

To ensure target dose coverage under density variation in the beam-paths due to breathing or DIBH variability, density overrides and more recently 4D robust optimisation are employed.^42^ The first method artificially increases the density of moving tissues while the second incorporates realistic instances such as multiple 4D phases or repeat DIBH scans in the optimisation. The success of these methods is measured with dose evaluations on multiple breathing phases (individually or deformed and accumulated into 4D dose) or breath-hold variations (Figure 6). Interplay effects between a moving target and a scanning beam are greatly reduced in DIBH treatments but need to be mitigated with empirical approaches in free breathing treatments. These approaches include setting motion upper limits for moving,^43^ rescanning, spot degradation, reduced beam modulation and decreased dose-rate. Several studies have shown that the use of combinations of these mitigation methods can average out the effect of interplay.^44–47^ To evaluate the effects of interplay and the applied mitigation strategies, 4D dynamic dose is calculated. These calculations not only include density variations based on 4D-CT phases but also incorporate timings of breathing and beam delivery.^48^

The additional uncertainties and the labour-intensive calculations to estimate the effects of motions make DIBH an appealing motion management for lymphomas.^49^ However, as pointed out earlier, treatment in DIBH does not necessarily result in reduced uncertainties. PBS therapy in DIBH comes with a complexity and other categories of uncertainties that needs to be understood. Slower deliveries, because of target size or machine delivery capabilities, require special considerations for the repeatability of large number of breath-holds. The effects of fatigue on breath-holds should be considered and mitigated. Newer techniques such the use of CPAP or oxygen-enhanced prolonged DIBH^50^ have the potential to alleviate aforementioned uncertainties but are not yet routinely used in PBT. It is important that each patient’s case and performance in the aspect of compliance and reproducibility is carefully evaluated to reach the robustness criteria. Discrepancies in dose distributions due to changes in breathing or breath-holds are identified on dose re-calculations on periodic or *ad-hoc* verification CT scans during the treatment course, which are common in PBT.

## Conclusion

Advances in technology have enabled a range of strategies to assess and minimise uncertainties in the delivery of RT. For patients treated for lymphomas and receiving mediastinal irradiation, these strategies have led to a considerable reduction in dose to OARs, such as the heart and the lungs. However, some gaps in knowledge remain. More work is needed to characterise intrafraction motion, both in DIBH and free breathing treatments. Newer technologies such as the MR-Linac, with the ability to acquire images during delivery without increasing the dose burden to the patient, may further contribute to this challenging scenario. Finally, it will be important to continue building the evidence base around the clinical impact of motion management strategies, irrespective of dosimetric benefits and quantify the benefits in terms of improved clinical outcomes and reduced toxicity.
